# Impact of Natural Organic Matter Competition on the Adsorptive Removal of Acetochlor and Metolachlor from Low-Specific UV Absorbance Surface Waters

**DOI:** 10.1021/acsomega.3c02588

**Published:** 2023-08-18

**Authors:** Emine Yilmaz, Ezgi Altiparmak, Filiz Dadaser-Celik, Nuray Ates

**Affiliations:** † Graduate School of Natural and Applied Sciences, Erciyes University, Kayseri 380320, Turkey; ‡ Department of Environmental Engineering, Erciyes University, Kayseri 380320, Turkey

## Abstract

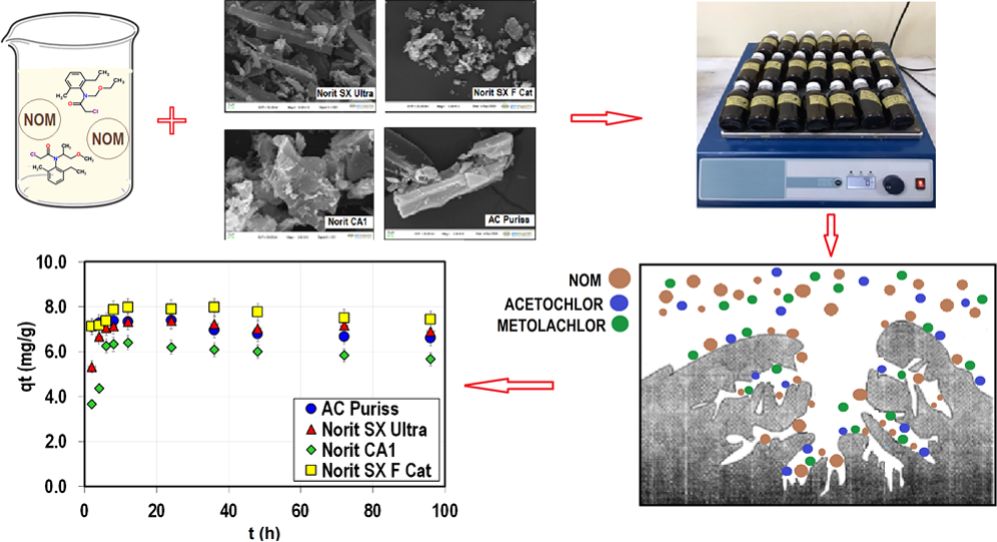

Although activated carbon adsorption is a very promising process for the removal of organic compounds from surface waters, the removal performance for nonionic pesticides could be adversely affected by co-occurring natural organic matter. Natural organic matter can compete with pesticides during the adsorption process, and the size of natural organic matter affects the removal of pesticides, as low-molecular-weight organics directly compete for adsorbent sites with pesticides. This study aims to investigate the competitive impact of low-molecular-weight organics on the adsorptive removal of acetochlor and metolachlor by four commercial powdered activated carbons. The adsorption features of selected powdered activated carbons were evaluated in surface water samples collected from the influent stream of the filtration process having 2.75 mg/L organic matter and 0.87 L/mg-m specific UV absorbance. The adsorption kinetics and capacities were examined by employing pseudo-first-order, pseudo-second-order, and intraparticle diffusion kinetic models and modified Freundlich and Langmuir isotherm models to the experimental data. The competitive removal of acetochlor and metolachlor in the presence of natural organic matter was evaluated for varied powdered activated carbon dosages on the basis of UV and specific UV absorbance values of adsorbed organic matter. The adsorption data were well represented by the modified Freundlich isotherm, as well as pseudo-second-order kinetics. The maximum organic matter adsorption capacities of the modified Freundlich isotherm were observed to be 120.6 and 127.2 mg/g by Norit SX Ultra and 99.5 and 100.6 mg/g by AC Puriss for acetochlor- and metolachlor-containing water samples, respectively. Among the four powdered activated carbons, Norit SX Ultra and AC Puriss provided the highest natural organic matter removal performances with 76 and 72% and 71 and 65% for acetochlor- and metolachlor-containing samples, respectively. Similarly, Norit SX Ultra and AC Puriss were very effective for adsorbing aromatic organics with higher than 80% specific UV absorbance removal efficiency. Metolachlor was almost completely removed by higher than 98% by Norit SX Ultra, Norit SX F Cat, and AC Puriss, even at low adsorbent dosages. However, an adsorbent dose of 100 mg/L and above should be added for all powdered activated carbons, except for Norit SX F Cat, for achieving an acetochlor removal performance of higher than 98%. The competition between low-molecular-weight organics (low-specific UV absorbance) and acetochlor and metolachlor was more apparent at low adsorbent dosages (10–75 mg/L).

## 1 Introduction

Pesticides are blends of organic and synthetic compounds, employed to combat insect pests, vectors, and weeds in household, industrial, agricultural, and other public areas^1^ as well as to increase the quality and quantity of products in agricultural activities.^2^ Pesticide concentrations in water resources are associated with crop type and density, as well as agricultural management techniques in the watershed.^3^ Previous studies on pesticide occurrence have shown that surface water resources in developing countries have substantially higher pesticide concentrations and carry a wider variety of pesticides than developed countries.^4^ Acetochlor and metolachlor, known as chloroacetanilides, are the most commonly used herbicides for corn, cotton, rice, soybean, sugar cane, sugar beet, and sunflower production to control broadleaf weeds and annual grasses. As they are relatively water-soluble, have lower volatility, are resistant to degradation, and are persistent in the environment, they are frequently identified in water and soil throughout the world.^5−7^ While acetochlor is listed as a B-2 carcinogen and a thyroid disrupter,^7^ metolachlor has toxic effects on the kidney, liver, and cardiovascular and nervous systems.^8,9^ It is anticipated that up to 96% of surface waters located close to agricultural activities contain detectable amounts of acetochlor as a result of transport and conversion mechanisms.^10^ In monitoring studies conducted in 38 rivers in the United States, 8 of the top 10 most detected organic chemicals were pesticides, including metolachlor.^11^

The first legislative action on pesticides, named the Federal Insecticide, Fungicide, and Rodenticide Act, was issued in 1947 in the United States,^12^ and federal standards were set for only 22 pollutants after updating the regulation in 1986 and 1996. This act required the nationwide monitoring of linuron, metolachlor, cyanazine, and metribuzin, among many pollutants. Three pesticides, including alachlor, metolachlor and their metabolites, and acetochlor, were included in the monitoring program in 2009.^13^ The European Council has identified 45 priority substances/substance groups in the Water Framework Directive based on research data on short- and long-term effects on the aquatic living organisms and human health, which include 12 organochlorine and 3 organophosphorus pesticides.^14^ The regulation of Surface Water Quality Management in Turkey defined Environmental Quality Standards for 250 pollutants; of which 45 were covered within the priority substance list. 19 pesticides were present in the list.^15^

Pesticide removal from water is primarily influenced by pesticide characteristics (solubility, polarity, volatility, acidity, lipophilicity, etc.), water characteristics (organic matter content, pH, type, and concentrations of background ions, temperature, etc.), and treatment techniques.^16^ For effective pesticide removal from water and wastewater, advanced treatment methods, such as adsorption, ion exchange, membrane filtration, advanced oxidation processes, and their hybrid combinations are recommended.^17−19^ Adsorption is considered a promising process for pesticide removal, since it is well-known, low-cost, and very effective. Several natural and commercial adsorbents are available as well as modified and functionalized adsorbents have been developed for removing toxic and micropollutants, including pesticides.^20−23^ Still, powdered activated carbon (PAC) remains the widely used adsorbent for pesticide removal because it is low-cost and easily available, it can be added when needed, its dosage can be adjusted easily according to pollutant concentration changes, it does not require a separate reactor, and it can be removed by precipitation or filtration processes after its use.^20^

The occurrence of natural organic matter in water sources can pose challenges to the effective removal of pesticides. It is considered that natural organic matter (NOM) in various molecular weights has different competitive impacts on micropollutant removal by the adsorption process from water sources, and in general, low-molecular-weight organics, which cannot be removed by coagulation, show the highest competition during micropollutant removal.^24−29^ The size of NOM affects the adsorption efficiency of micropollutants by decreasing the adsorbent capacity. While low-molecular weight NOM compete for direct sites, high-molecular weight NOM cause pore entrance blockage. In the removal of micropollutants by activated carbon adsorption, the dominant competitor for the active sites of the adsorbent is NOM with a molecular weight of less than 600 g/mol in natural waters, and low-molecular-weight organics compete with micropollutants by blockage of the micropores of the adsorbent. On the other hand, the effects of competitive and pore blockage of NOM might be less significant for mesoporous-activated carbons.^30^ Depending on the characteristic of NOM specific to the water source and the pore size distribution and size of activated carbon, the degree of the competitive effect of NOM against micropollutants can be different. Specific UV absorbance (SUVA) represents the numerical quantity of aromatic content and the humic fraction, since the aromatic structure of NOM containing conjugated C=C double bonds absorbs UV light at 254 nm. SUVA normalized by dividing UV_254_ by the dissolved organic carbon (DOC) value is widely used as a surrogate parameter of predominant organic compounds for water-containing NOM mixtures.^31,32^ When the SUVA value is lower than 2 L/mg-m, water sources in terms of NOM are defined as having non-humic, mostly aliphatic, lower-molecular-weight fractions and hydrophilic compounds and are characterized as low-SUVA waters.^33−35^

There are a few studies on the removal of acetochlor and metolachlor from water and wastewater by activated carbon using synthetic model waters or real waters. In the majority of these studies, acetochlor or metolachlor concentrations ranged between 10 and 100 mg/L, much higher than those expected in surface waters.^36−40^ Although competition between NOM and pesticides would be expected in the adsorption process, only few studies investigated the impact of organic characteristics on the nonionic pesticide (e.g., acetochlor and metolachlor) removal mechanism.^26−28^ The main objective of this study is to investigate the competitive removal of acetochlor and metolachlor from surface waters in the presence of low-molecular-weight NOM with hydrophilic characteristics by four different powdered activated carbons (PACs). The water samples used in this study were collected from the influent stream of the filtration process and had 2.75 mg/L NOM (in terms of DOC), 0.024 cm^–1^ UV_254_, and 0.87 L/mg-m SUVA. Among the PACs, one was steam-modified and the other one was chemically modified. The other two were untreated. In the scope of the study, the adsorption kinetics and capacities for selected PACs were determined as well as the removal performances of acetochlor, metolachlor, and NOM were evaluated.

## 2 Material and Methods

### 2.1 Water Samples and Chemicals

The water samples were collected from the influent stream of the filtration process in the Konya Water Treatment Plant (Turkey) in April 2021. The Konya Water Treatment Plant receives water from the Altinapa Reservoir and supplies drinking water to the city of Konya with 2.5 million population. In previous studies, acetochlor and metolachlor were identified at levels higher than the Environmental Quality Standards provided in the regulation of Surface Water Quality Management in Turkey.^15,41^ Water samples collected were carried to the laboratory in 25 L cooled jerricans and kept at +4 °C in a fridge for experimental studies. The properties of the water samples are presented in Table 1.

**Table 1:** Physicochemical Properties of Water Samples

parameter	value^a^
pH	7.85
electrical conductivity (μS/cm)	367
total hardness (mgCaCO_3_/L)	210
alkalinity (mgCaCO_3_/L)	201
UV_254_ (1/cm)	0.024
dissolved organic carbon (DOC) (mg/L)	2.75
total nitrogen (TN) (mg/L)	1.50
specific UV absorbance (SUVA) (L/mg-m)	0.87

a The values for parameters are the average of three measurements.

Acetochlor (Cat. No. Supelco 33379) and metolachlor (Cat. No. Supelco 36163) analytical standards (≤100% purity) were supplied from Sigma-Aldrich. The major properties of the tested pesticides are shown in Table 2. The chemicals (chloroform, acetone, methanol, and acetonitrile) used in pesticide analysis were HPLC/GC grade and obtained from Merck (Merck, Darmstadt, Germany).

**Table 2:**
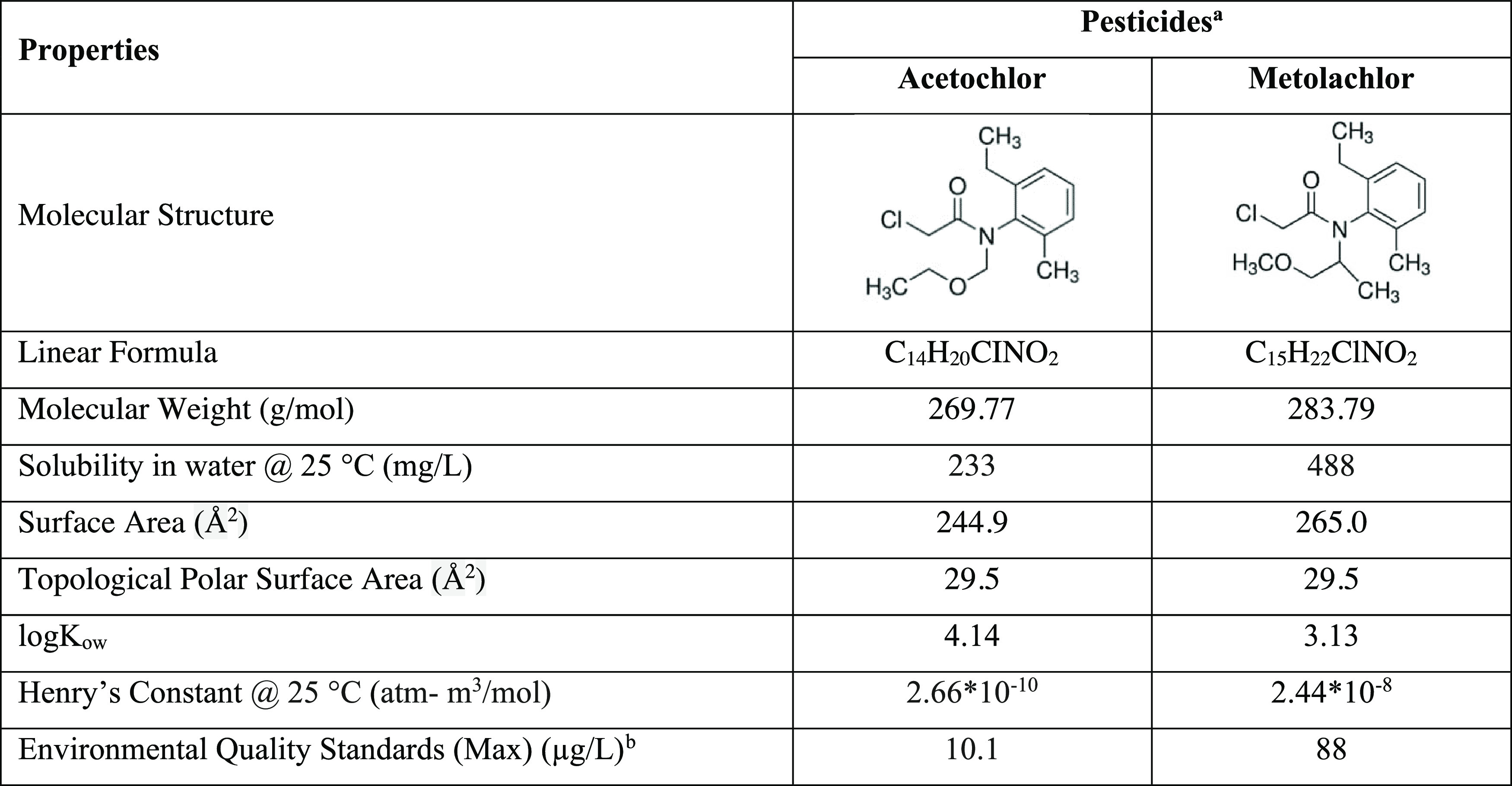
Properties of Acetochlor and Metolachlor

a The properties of pesticides were provided from https://pubchem.ncbi.nlm.nih.gov/.^42^

b The regulation of Surface Water Quality Management in Turkey.

### 2.2 Adsorbents

In adsorption experiments, four different PACs, Norit SX Ultra (Cat. No. 53663), Norit CA1 (Cat. No. 97876), Norit SX F Cat (Cat. No. 901933), and AC Puriss (Cat. No. 31616), obtained from Sigma-Aldrich, were used. While Norit SX Ultra and Norit CA1 were originally steam- and chemically modified, respectively, the other two activated carbons were originally untreated. The adsorbents were characterized in terms of particle size distribution, surface chemistry (point of zero charge (pH_pzc_), total acidic and basic groups, functional groups), surface area, and morphology.

### 2.3 Experimental Procedure: Kinetic and Isotherm Adsorption Tests

Adsorption testing was performed in two stages: (1) kinetic tests were applied to determine the time to equilibrium and (2) equilibrium tests were applied to assess adsorption capacities. Kinetic tests were accomplished at a 300 mg/L constant adsorbent dose in 100 mL of water samples in amber glass bottles with a PTFE cap in two sets containing 500 μg/L either acetochlor or metolachlor. In order to identify the equilibrium time, the samples were thoroughly mixed at 120 rpm on a shaker in a horizontal position for 2, 4, 8, 12, 24, 36, 48, and 72 h. In equilibrium experiments, 100 mL of water samples, to which PACs were added at 10 different doses, ranging from 10 to 1000 mg/L, were prepared in two sets containing 500 μg/L either acetochlor or metolachlor and were thoroughly shaken horizontally at 120 rpm at a temperature of 25 ± 2 °C. The pHs of all samples were set to 8 ± 0.1 with K_2_HPO_4_ and KH_2_PO_4_ buffer solutions. After a certain contact time, which had been previously determined in kinetic tests, the PACs were separated from samples by 0.45 μm filter paper and kept in the fridge until further analytical tests. The filter paper had previously been washed with distilled water (500 mL) to avoid possible organic leaks from the filter. Acetochlor, metolachlor, DOC, UV absorbance, pH, and conductivity analyses were employed on water samples obtained from kinetic and isotherm tests. All tests were conducted in two parallels; all parameters were analyzed three times, and the averaged data were presented. Control samples without activated carbon were also included in all isotherm experiments. DOC values were used to evaluate kinetic types and rates and adsorption capacities of PACs, since the NOM content of the samples was relatively higher, and they had higher competition against adsorbents than pesticides.

The adsorption capacity (NOM absorbed on PACs) was calculated by dividing the difference between the initial and final adsorbate concentrations by the adsorbent dose, given in eq 1.
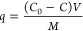
1In eq 1, *C*_0_ and *C* represent the initial and final quantities of organic matter in the aqueous phase (mg/L), respectively. Adsorption capacity (mg/g), sample volume (L), and mass of PACs (g) are indicated by *q*, *V*, and *M*, respectively.

The linear forms of three common kinetic models, including the pseudo-first-order model (PFO), pseudo-second-order model (PSO), and intraparticle diffusion model (IPDM), were adapted to the kinetic data. The linearized PFO and PSO models are given in eqs 2 and 3.
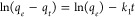
2
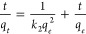
3In eqs 2 and 3, *k*_1_ (1/h) and *k*_2_ (g/mg-h) are the rate constants of PFO and PSO kinetic models, respectively; *q*_*t*_ and *q*_*e*_ are the adsorption capacities (mg/g) at time *t* and equilibrium, respectively.

The IPDM, proposed by Weber and Morris,^43^ has been extensively applied to interpret the rate-limiting step of the adsorption process and defined as follows (eq 4)
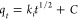
4Here, *q*_*t*_ is the adsorption capacity at time *t* (mg/g), *k*_*i*_ is the pore diffusion parameter (mg/g-h^1/2^), *t* is the contact time, and *C* is an arbitrary constant (mg/g).

### 2.4 Analytical Methods

Acetochlor and metolachlor were analyzed by a Shimadzu LC-2030C HPLC, equipped with a GL Sciences Inertsil ODS-4 (particle size: 5 μm, length: 250 mm, i.d: 4.6 mm column, GL Sciences), and a UV detector. Calibration standards and water samples containing acetochlor and metolachlor were extracted using the dispersive liquid–liquid microextraction (DLLME) technique before HPLC analysis. For the quantitative analysis of pesticides, an 8-point calibration curve was used using standard samples prepared from acetochlor and metolachlor standard mixtures. In the extraction procedure, 1,2-dichloroethane (400 μL) and acetonitrile (1 mL) extraction–dispersive solvent mixtures for acetochlor and 1,2-dichloroethane (300 μL) and methanol (1 mL) extraction–dispersive solvent mixtures for metolachlor were added to either calibration solutions or water samples (8 mL). In the extraction procedure, the samples were first vortexed for 1 min and later centrifuged at 6000 rpm for 2.0 min, and finally, the separated organic phases were taken into vials containing 100 μL inserts to be analyzed by the HPLC instrument at wavelengths of 210 nm for acetochlor and 230 nm for metolachlor. The limit of detection (LoD) and limit of quantification (LoQ) levels were 0.89 and 2.71 μg/L for acetochlor and 1.17 and 3.54 μg/L for metolachlor, respectively. The amount of NOM in terms of DOC was determined according to SM 5310 B^44^ with a TOC analyzer (TOC-L CPH, Shimadzu) after the samples were filtered through a 0.45 μm filter. A-6 point calibration standards were prepared for concentrations ranging from 0.2 to 6 mg/L with potassium hydrogen phthalate. A UV–visible spectrophotometer (Hach Lange DR 6000) was used to measure the UV absorbance of water samples at a wavelength of 254 nm according to the SM 5910 B method.^44^ Conductivity (SM 2510) and pH were measured (SM 4500-H^+^) by a Hach (HQ40d) multimeter.

The surface chemistry of PACs is characterized by acid and base neutralization capacities, which are neutral charge points (pH_pzc_). The pH_pzc_ values of the activated carbon samples were attained in compliance with the method of Dastgheib et al.^45^ The pH values of a 0.1 M NaCl solution prepared in pure water were adjusted in 2, 3, 4, 5, 6, 7, 8, 9, 10, 11, and 12 with 0.5 M HCl and/or 0.5 M NaOH. 20 mL of the sample was mixed by adding 100 mg of the adsorbent at 100 rpm and 20 ± 5 °C for 48 h. The pH_pzc_ value of the samples is equal to the initial pH value, and no change is observed during the contact period after the adsorbent is added. Total surface acidic and total surface basic groups were determined by applying the Boehm method (alkalimetric titration) with minor modification of modifications in the method by Dastgheib et al.^45^ A series of 20 mL of 0.05 N NaOH or 0.05 N HCl solutions containing 200 mg of adsorbents were mixed at room temperature at 100 rpm for 48 h. Additional blank samples without adsorbents were also included in the test. At the end of contact time, the samples were left for 4 h for precipitation of the adsorbents and then filtered. 10 mL of the filtered samples were titrated with either 0.05 N HCl or 0.05 N NaOH. The differences in the spent amount of HCl/NaOH for the blank and adsorbent-containing samples were used to calculate the total surface acidic and basic groups.

Surface areas (BET) and total pore volumes of the adsorbents were determined with a Micromeritics Gemini VII Surface Area and Porosity Surface Analyzer. FTIR analyses of adsorbents were carried out by a Perkin Elmer 400 FT-IR/FT-FIR device in the wavelength range of 400–4000 cm^–1^. The particle size distribution of the adsorbents was determined by dynamic light scattering (DLS) analysis. DLS parameters of PACs were determined using a Malvern NanoZS90 instrument equipped with a 633 nm laser at ambient temperature. Data were collected with a scattering angle of 173°. BET, FTIR, and DLS analyses were performed at the Erciyes University Technology Research and Application Center.

## 3 Results and Discussion

### 3.1 Characterization of Adsorbents

**Figure 1:**
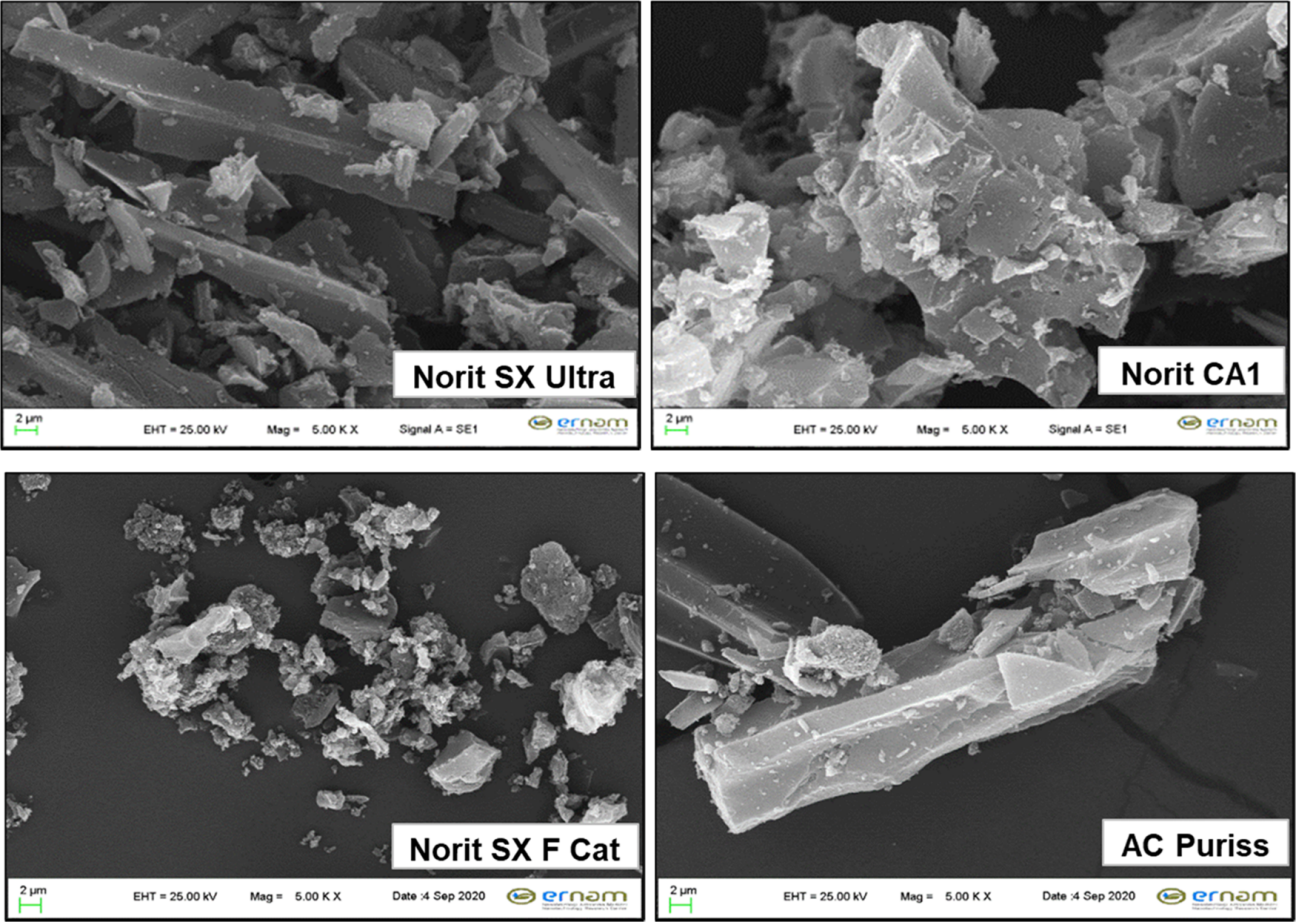
SEM images of activated carbons (2 μm mag: 500k×).

Particle sizes of the adsorbents were determined by the weighted average calculation method using DLS histograms. The particle sizes of Norit SX Ultra were distributed between 190 and 1718 nm, with a mean size of 379 nm. Approximately 80% of the particles had particle sizes within the range of 190 and 396 nm. The mean particle size of Norit CA1 was 685 nm, and about 80% of particles were between 396 and 955 nm. Although the average particle size of Norit SX F Cat was 938 nm (255–6439 nm), about 55% of the particles had sizes below 955 nm. On the other hand, AC Puriss had an average particle size of 253 nm and its particle sizes ranged from 122 to 3091 nm, while 85% of the particles were smaller than 295 nm. The SEM images of the adsorbents at 2 μm size and a 500k× magnification scale are given in Figure 1. For all PACs, the apparent porosity can be seen from images. The particles of Norit SX Ultra and AC Puriss were mostly in finger shape, while the particles of Norit CA1 and Norit SX F Cat were in distorted spherical form. Besides, the particle size distribution of Norit SX Ultra and Norit SX F Cat had a wider range, which can also be seen in SEM images.

**Figure 2:**
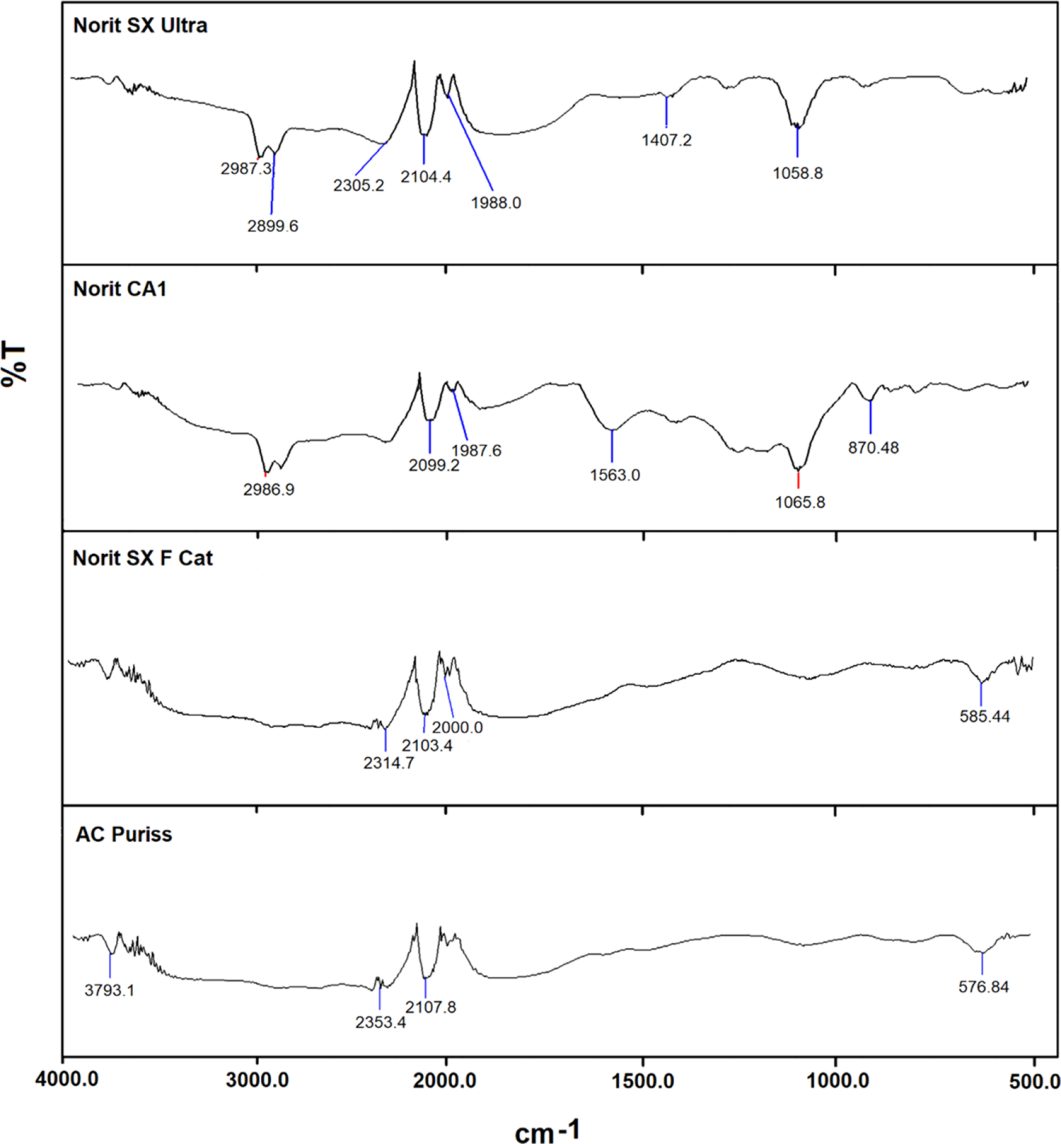
FTIR spectra of tested PACs.

The FTIR spectra for Norit SX Ultra, Norit CA1, Norit SX F Cat, and AC Puriss are presented in Figure 2. There are three and four major bands observed for Norit SX F Cat and AC Puriss adsorbents, respectively. The transmission in the range of 450–750 cm^–1^ is attributed to the aromatic ring deformation, and 585 and 576 cm^–1^ absorption bands correspond to C–H bending mode.^46^ The vibrations at 2000 cm^–1^ correspond to the C=C=N stretch of ketenimine; those at 2103 and 2107 cm^–1^ indicate the C≡C stretch of alkyne groups, and the signals at 2314 and 2353 cm^–1^ may represent ketone groups and the vibration of the −COOH structure for Norit SX F Cat and AC Puriss adsorbents.^47−49^

On the other hand, six signals were detected for Norit SX Ultra and Norit CA1. The vibration at 870 cm^–1^ indicates the existence of strong C–H bending. The presence of C–O stretching vibrations in alcohols, phenols, acids, ethers, or esters is indicated by the absorption at 1058 and 1065 cm^–1^. While the 1407 cm^–1^ absorption band can be attributed to the O–H bond of carboxylic acid or alcohol^50^ and O=C–O belonging to the −COOH groups.^7,51^ The C=C stretching vibration in aromatic rings, specifically containing NO_2_ groups, is responsible for the peak at 1563 cm^–1^.^47,52^ The C=C=N stretch of ketenimine groups was observed between 1987 and 1988 cm^–1^. The symmetric and asymmetric C–H stretching vibrations of aliphatic acids are supposed to be responsible for the peaks of 2899, 2986, and 2987 cm^–1^.^53^

The average BET area, total pore volume, pore diameter, pH_pzc_, and total acidic and basic groups of the adsorbents are given in Table 3. The surface areas and pore sizes of the adsorbents were attained from N_2_ adsorption–desorption isotherms based on the nitrogen (N_2_) gas adsorption technique in a liquid nitrogen environment at 77 K. The N_2_ adsorption–desorption isotherms of all adsorbents more likely conform to the Type IV isotherm curve of mesoporous materials according to the IUPAC classification, which represents the layer-by-layer adsorption of mesoporous materials on a smooth non-porous surface. While Norit SX Ultra and Norit CA1 had similar BET average surface areas of 1200 and 1161 m^2^/g, respectively, AC Puriss had the lowest surface area of 274 m^2^/g.

**Table 3:** Physical and Chemical Characteristics of Activated Carbons

adsorbent		Norit SX Ultra	Norit CA1	Norit SX F Cat	AC Puriss
BET surface area *S*_BET_ (m^2^/g)		1200	1161	657	274
total pore volume (cm^3^/g)		0.95	1.12	0.78	0.22
average pore diameter (nm)		3.16	3.86	4.75	3.24
pH_PZC_		8.11	2.53	7.8	7.09
total acidic groups	(meq/g)	3.6	4.0	3.5	3.8
	(meq/m^2^)	0.003	0.003	0.005	0.014
total basic groups	(meq/g)	3.33	3.03	3.8	3.9
	(meq/m^2^)	0.003	0.003	0.006	0.014

The pH_pzc_ value shows that the adsorbent surface would be positively or negatively charged based on the pH of the water samples. At pH values lower than pH_pzc_, the surface of the adsorbent is mostly positively charged, while at higher pH values, it is negatively charged. As shown in Table 3, with a pH_pzc_ value of 7.8, the surface of Norit SX F Cat was neutral, since the pH of the water sample was 7.85 (Table 1). The pH_pzc_ values for Norit SX Ultra and AC Puriss were determined as 8.11 and 7.09, implying that the surfaces of adsorbents were slightly positively and negatively charged in the solution, respectively. On the other hand, the surface of Norit CA1, having the lowest pH_pzc_ value of 2.53 (pH ≫ pH_pzc_), would be strongly negatively charged. The strong O–H stretching of 2986.9 cm^–1^ belonging to carboxyl groups and strong C–O stretching of 1065.8 cm^–1^ belonging to primary alcohols might be responsible for the negatively charged surface of Norit CA1.^48,54^ The amount of total surface acidic and basic groups identified by the Boehm method also supported this phenomenon that having higher acidic groups resulted in lower pH_pzc_ values.^55^

### 3.2 Effect of Contact Time

**Figure 3:**
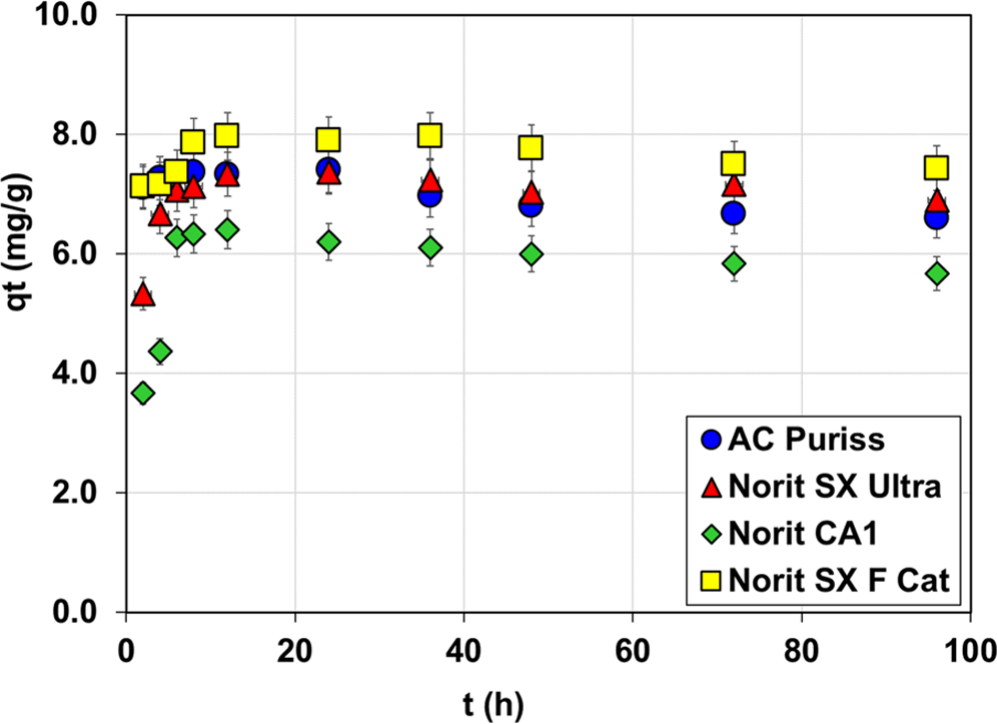
Adsorption capacities of PACs versus time (pH: 7.85, *C*_0_: 2.75 mg/L, *T*: 25 °C, m: 300 mg/L, stirring at 120 rpm).

The equilibrium times for organic matter (in terms of DOC) adsorption onto activated carbons were evaluated, and the results are presented in Figure 3. As mentioned earlier in Section 2, since the concentrations of acetochlor and metolachlor in the samples are very low compared to the amount of organic matter and there is significant competition between them, the adsorption kinetic and isotherm models were investigated on the basis of organic matter. Adsorption of organic matter by adsorbents occurred very rapidly in the first few hours within the 96 h contact time. Especially AC Puriss and Norit SX F Cat carbons reached the equilibrium plateau within the first 6 h. On the other hand, the equilibrium plateau for Norit SX Ultra and Norit CA1 carbons was gradually attained after 10–12 h. When the adsorption equilibrium phase was reached, the lowest and the highest DOC removal performances were obtained with 60 and 75% on average by the Norit CA1 and Norit SX F Cat activated carbons, respectively. Besides, on average, 70% DOC removal was observed for AC Puriss and Norit SX Ultra adsorbents. In previous studies, the time for adsorbents to reach equilibrium in the removal of organic materials and/or pesticides with activated carbon is identified as between 0.5 h and 7 days.^56,57^

Adsorption of NOM by PAC is a complex phenomenon, as it is a complex heterogeneous combination of humic acids, fulvic acids, low-molecular-weight organic acids, carbohydrates, proteins, and other components. Therefore, different mechanisms such as hydrophobic effect, electrostatic interactions, hydrogen bonding, and π–π bonds are effective during the adsorption of NOM on the PAC in the aqueous phase.^58^ Electrostatic interactions predominate in adsorbing NOM, which is generally negatively charged in surface waters, by positively charged PAC; on the other hand, the pore size of adsorbents is the primary control factor for NOM adsorption.^59^ As seen from Figure 3, all PACs provided similar adsorption behavior in terms of NOM adsorption from aqueous solution, even though they have a wide range of pH_pzc_ values (2.53–8.11). However, the slower adsorption rate (in early contact times) and capacity of Norit CA1 with respect to others is due to its strongly negative charges repelling NOM compounds. Norit SX F Cat and AC Puriss were neutral or slightly negatively charged at 7.85 pH of the water sample, and it was assumed that their pore sizes of 4.75 and 3.24 nm were primary control factors for NOM adsorption, respectively. Besides, the oxygen-containing functional groups on the structure of Norit SX F Cat and AC Puriss might have a dominant role by making H-bonds between NOM moieties and adsorbents.^58^

### 3.3 Adsorption Kinetic Models

**Figure 4:**
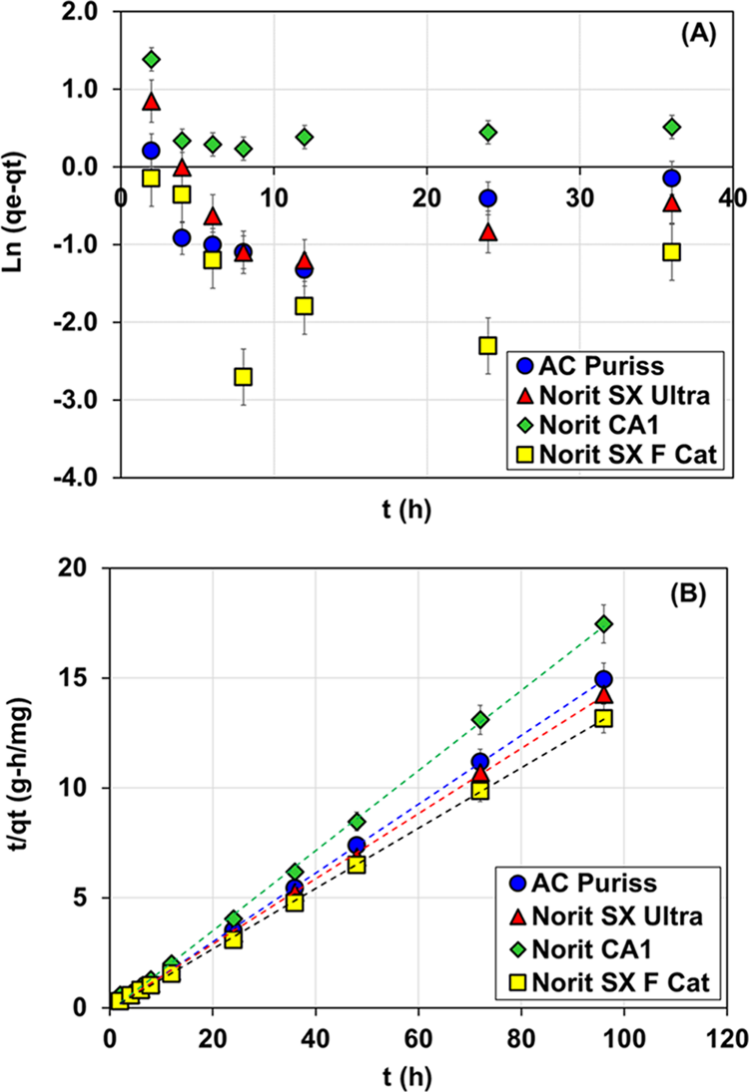
Pseudo-first-order kinetic (A) and pseudo-second-order kinetic (B) models for NOM adsorption by tested PACs (pH: 7.85, *C*_0_: 2.75 mg/L, *T*: 25 °C, m: 300 mg/L, stirring at 120 rpm).

Since their accurate depiction of the actual data, pseudo-kinetic models, which simulate the total rate of adsorption, are commonly used for modeling the kinetics of adsorption processes.^60,61^ The PFO plot with ln(*q_e_* – *q_t_*) plotted against t and the PSO plot with *t*/*q_t_* plotted against t for determining the organic matter adsorption kinetic rates are shown in Figure 4. The kinetic results obtained from the water samples containing acetochlor and metolachlor were relatively similar, and since the differences between kinetic rates and coefficients for both models were less than 5%, the average results for the kinetic parameters for these two data sets were given. The data from the kinetic experiments were better explained by PSO, having a higher *R*^2^ with 0.99 for all activated carbons, as shown in Table 4. In addition, the predicted equilibrium capacity for the PSO kinetic model (*q*_*e*,calc_) corresponded more closely to the experimental equilibrium capacity (*q*_*e*,exp_) than it was for the PFO kinetic model (Table 4). Based on the PSO model results, it might be concluded that the ratio between the square of the number of vacant sites on the adsorbents and the occupancy rate of adsorption sites was linear.^62^ In addition, the better fit of data to the PSO model than that to the PFO model can be attributed to chemical absorption processes, such as hydrogen bonds and π–π interactions, which may be more dominant in the adsorption of organic matter to the adsorbent.^7,63,64^

**Table 4:** Adsorption Kinetic Model Parameters for NOM Adsorbed on Tested PACs

	pseudo-first-order				pseudo-second-order			
adsorbents	*q_e_*_,exp_ (mg/g)	*q_e_*_,cal_ (mg/g)	*k*_1_ (1/h)	*R*^2^	*q_e_*_,cal_ (mg/g)	*k*_2_ (g/mg-h)	*R*^2^	*H*
Norit SX Ultra	6.82	2.41	0.20	0.85	6.75	0.39	0.99	17.83
Norit CA1	5.56	1.36	0.005	0.85	5.49	0.24	0.99	7.32
Norit SX F Cat	7.33	10.83	0.02	0.74	7.28	0.25	0.99	13.42
AC Puriss	6.46	1.02	0.13	0.66	6.39	0.19	0.99	7.96

The PSO rate constant (*k*_2_) and initial sorption rate (*H*) for the adsorbents tested in this study displayed various ordering. The rate constants (*k*_2_) of the PSO model and the initial sorption rate constants (*H*) followed the order of Norit SX Ultra > Norit SX F Cat ≥ Norit CA1 > AC Puriss and Norit SX Ultra > Norit SX F Cat > AC Puriss ≥ Norit CA1, respectively. Indeed, Norit SX Ultra and Norit CA1 were originally steam- and chemically modified activated carbons, respectively, while the others were not modified. Although Norit CA1 was chemically modified, its initial adsorption rate and PSO rate constant were lower than those of Norit SX F Cat, which is an unmodified adsorbent. This can be attributed to its repulsion of organics in solution with a pH of 7.85, as it has a pH_pzc_ of 2.54, leading to a negatively charged surface.^65−67^ On the other hand, the lower PSO rate constant and initial rate observed are thought to be related to the very low surface area of AC Puriss compared to other activated carbons.

**Figure 5:**
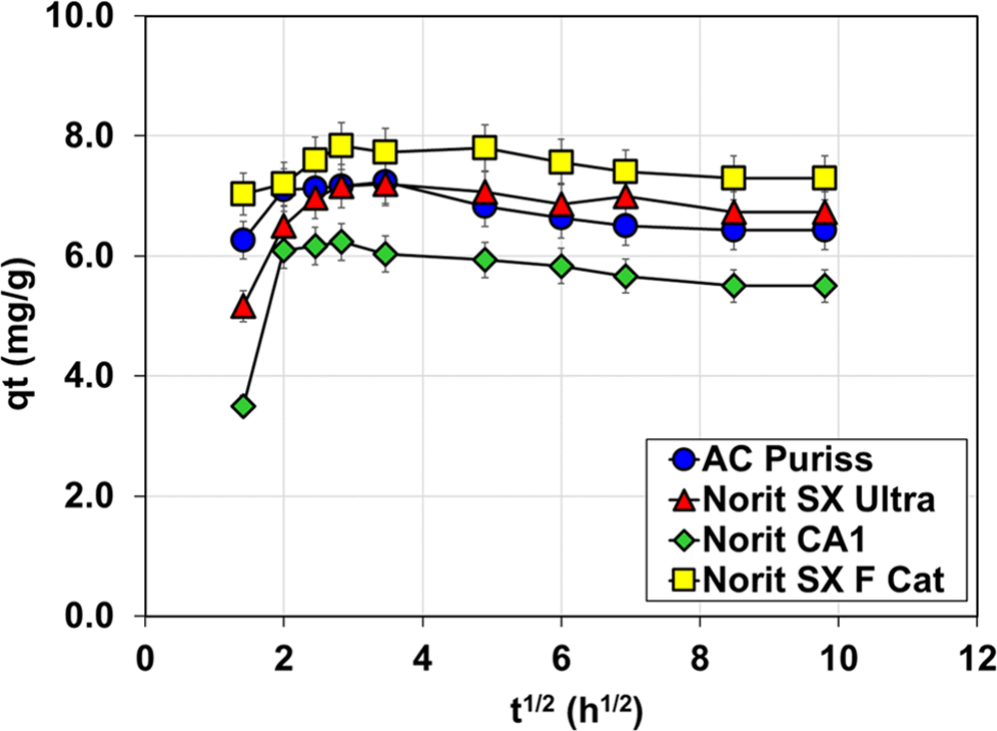
Weber–Morris model for the adsorption kinetics of NOM on the tested carbons.

The sorption of organic matter onto activated carbon is a complicated process, in which the characteristics of both the absorbent and the adsorbate are important. The three sequential steps that can be applied to control the adsorption process are (1) bulk solution transport, (2) film diffusion, and (3) pore diffusion and adsorption, in which the adsorbate is transported to available adsorption sites within the pores of activated carbon. The adsorption process may involve one or more of these stages; the one with the slowest rate determines how much material is absorbed.^68,69^ Pore diffusion should exhibit a straight line with a slope equal to *k_i_* (shown in eq 4) if it is the rate-limiting phase in the adsorption process. Pore diffusion plots frequently display many linear segments; therefore, the procedure cannot be that straightforward in practice.^70^ These linear segments may reflect pore diffusion in pores with progressively smaller sizes. The IPDM results are given in Figure 5, and the adsorption of organic matter onto activated carbons consisting of basically two steps was attributed to possibly rapid intraparticle diffusion.^62,71,72^ As seen from the figure, the plots were nonlinear and did not cross the origin; it may be inferred that the chemical reaction or film diffusion is the limiting step.^69^ Further, since the plots of *q_t_* against *t*^1/2^ did not yield a straight line, the parameters for the interparticle diffusion model could not be determined.

### 3.4 Adsorption Equilibrium Models

Two widely used empirical isothermal adsorption models, Langmuir and Freundlich, were applied to fit the equilibrium adsorption data of DOC at different adsorbent doses in order to determine the affinity of equilibrium organic matter adsorption onto selected activated carbons. The Langmuir model assumes that there is a uniform surface having a fixed number of sites, and only one solute molecule is adsorbed per site. On the other hand, the assumptions for the Freundlich isotherm are the occurrence of adsorbate uptake on a heterogeneous surface by multilayer adsorption and the increase of the amount of the adsorbate adsorbed infinitely with the increase in concentration.^73−75^ The modified Freundlich isotherm model is used to define the adsorption isotherm of multicomponent and unknown organic mixtures with different adsorption affinities and to better compare the measured adsorption parameters (DOC and UV_254_) with the predicted ones.^76−78^ In this study, dose-base the dose-based normalized modified Freundlich isotherm model was applied to experimental data. The goodness of fit of data to linearized Langmuir and modified Freundlich models was evaluated using eqs 5 and 6, respectively
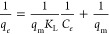
5
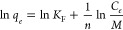
6The separation factor was calculated as follows (eq 7).
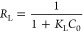
7In these equations, *q*_*e*_ and *q*_m_ indicate the amounts adsorbed at equilibrium and the maximum adsorption capacity of the adsorbent (mg/g), respectively. *K*_L_ and *K*_F_ are the adsorption constants at equilibrium for Langmuir and modified Freundlich isotherm models, respectively. 1/*n* denotes the adsorbent surface affinity varying with the heterogeneity of surface site energy distribution. *M* is the adsorbent dosage. *R*_L_ is the separation factor representing the Langmuir isotherm feature, and *C*_0_ is the initial adsorbate concentration.

Isotherm model parameters obtained as a result of linearized analysis of Langmuir (1/*q*_e_ vs 1/*C*_e_) and modified Freundlich (log *q*_e_ vs log *C*_e_/dose) isotherm models are given in Tables 5 and 6, respectively. The modified Freundlich isotherm better fitted the organic matter removal data for all adsorbents than the Langmuir isotherms, with correlation coefficients of 0.98–0.99 (*R*^2^). The weaker fit of the experimental data to the Langmuir isotherm model was observed, as high adsorbate concentrations at equilibrium predicted low adsorption capacity. The Langmuir model is generally not suitable for adsorption in the case of a high concentration of the adsorbate at equilibrium, since the Langmuir isotherm model disrupts the assumptions of single-layer coverage, site equivalence, and site independence.^68^

**Table 5:** Isotherm Parameters of the Langmuir Model for NOM Adsorption in Water Samples Containing Acetochlor and Metolachlor^a^

	acetochlor				metolachlor			
adsorbents	*q*_m_	*K*_L_	*R*_L_	*R*^2^	*q*_m_	*K*_L_	*R*_L_	*R^2^*
Norit SX Ultra	2.53	0.57	0.36	0.90	2.94	0.59	0.36	0.79
Norit CA1	2.34	0.48	0.41	0.82	2.0	0.44	0.43	0.86
Norit SX F Cat	2.24	0.55	0.39	0.81	2.57	0.56	0.38	0.83
AC Puriss	3.16	0.57	0.36	0.60	3.43	0.68	0.39	0.65

a The units of *q*_m_ and *K*_L_ are mg/g and (L/mg), respectively.

**Table 6:** Isotherm Parameters of the Modified Freundlich Model for NOM Adsorption in Water Samples Containing Acetochlor and Metolachlor

	acetochlor				metolachlor			
adsorbents	*q*_e,max_ (mg/g)	*K*_F_^a^	1/*n*	*R*^2^	*q*_e,max_ (mg/g)	*K*_F_^a^	1/*n*	*R*^2^
Norit SX Ultra	120.6	2.50	0.74	0.99	127.2	2.18	0.79	0.99
Norit CA1	86.7	1.84	0.70	0.98	76.7	1.64	0.72	0.98
Norit SX F Cat	91.1	1.70	0.76	0.98	93.5	1.63	0.79	0.98
AC Puriss	99.5	2.58	0.72	0.98	100.6	1.89	0.79	0.99

a The unit of *K*_F_ is (mg/g)/(mg/L)^1/*n*^.

**Figure 6:**
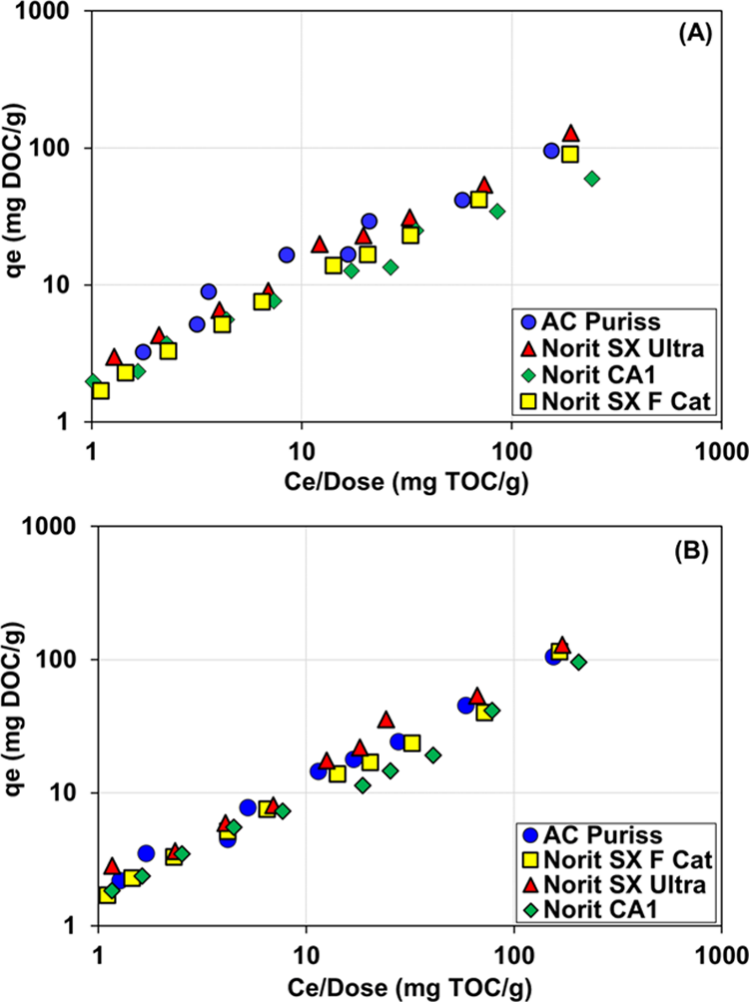
Dose-normalized equilibrium of modified Freundlich isotherm of organic matter by tested activated carbons: (A) Acetochlor-containing samples and (B) metolachlor-containing samples (pH: 7.85, *C*_0_: 2.75 mg/L, *T*: 25 °C, stirring at 120 rpm).

The dose-based linearized modified Freundlich isotherm for organic matter adsorption from acetochlor- and metolachlor-containing water samples is depicted in Figure 6A,B, respectively. 1/*n*, a measure of adsorption density or surface heterogeneity, is the slope of the modified isotherm plot and ranges from 0 to 1; the closer the value to 0, the greater the heterogeneity.^79^ The values of 1/*n*, obtained from the modified Freundlich isotherm for acetochlor- and metolachlor-containing water samples, vary from 0.70 to 0.76 and from 0.72 to 0.79, respectively, indicating that the adsorption of organic matter by all tested activated carbons was favorable and suggested an irreversible chemisorption process (1/*n* < 1) as reported in the study of adsorption of organic matter by powdered activated carbon by many researchers.^75,80−82^

The characteristic of an isotherm is defined by the separation factor of *R*_L_, which indicates whether an isotherm is irreversible (*R*_L_ = 0), favorable (0 < *R*_L_ < 1), linear (*R*_L_ = 1), or unfavorable (*R*_L_ > 1).^73,75,83^ Further, the separation factor of *R*_L_ values from the Langmuir isotherm ranging between 0.36 and 0.43 for the acetochlor- and metolachlor-containing water samples also supported the favorable adsorption of organic matter by selected adsorbents.^73,83,84^ On the other hand, it can be deduced that the adsorption density is weak, and the surface heterogeneity of activated carbon is a less significant factor, since the 1/*n* values obtained in experimental studies were close to 1.^65,73,85^

Among the activated carbons, Norit SX Ultra yielded the highest adsorption density with 1/*n* values of 0.74 and 0.79 for the acetochlor and metolachlor samples, respectively. The *K*_F_ values range from 1.70 to 2.58 (mg/g)/(mg/L)^1/*n*^ for the acetochlor samples and from 1.63 to 2.18 (mg/g)/(mg/L)^1/*n*^ for the metolachlor samples. The highest *K*_F_ values of 2.20 and 2.58 (mg/g)/(mg/L)^1/*n*^ for the acetochlor samples and 2.18 and 1.89 (mg/g)/(mg/L)^1/*n*^ for the metolachlor samples revealed stronger adsorption of organic matter onto Norit SX Ultra and AC Puriss, respectively. Similar to the highest affinity (*K*_F_) observed between the adsorbent and the adsorbate, the maximum modified Freundlich isotherm capacities of 120.6 and 99.5 mg/g for acetochlor and 127.2 and 100.6 mg/g for metolachlor were obtained by Norit SX Ultra and AC Puriss, respectively. Similarly, Lu and Su^86^ determined the Freundlich isotherm parameters of K_F_ and 1/*n* as 3.86 and 0.63, and Iriarte-Velasco et al.^67^ reported them as 5.85 and 0.63 for organic matter removal by activated carbon, respectively. Interestingly, although Norit CA1 was chemically modified and had a higher surface area than Norit SX F Cat and AC Puriss, the lowest affinity values and the lowest capacity were provided by this activated carbon. The main reason for this is attributed to the fact that Norit CA1 is negatively charged with a pH_pzc_ value of 2.53 and repels organic compounds. On the other hand, AC Puriss has the lowest surface area; however, it has the highest number of total basic groups (3.9 meq/g), which plays a significant impact on the adsorption of organic compounds.^87^

### 3.5 Effect of Adsorbent Dose on the Removal of Acetochlor, Metolachlor, and NOM

The removal performances of tested activated carbons for acetochlor, metolachlor, and NOM were evaluated by varying doses from 10 to 1000 mg/L in completely mixed batch reactors at an initial pH of 7.85, a mixing speed of 120 rpm, and a temperature of 25 °C. The surface water samples contained 500 mg/L either acetochlor or metolachlor and 2.75 mg/L NOM. In Table 7, the removal efficiencies of acetochlor, metolachlor, and NOM were presented. Besides, the removal percentages of UV_254_ absorption and SUVA were determined to evaluate the impact of adsorption on the NOM fraction.

**Table 7:** Effect of the Adsorbent Dose on the Removal of Acetochlor, Metolachlor, and NOM (%)^a^

	Norit SX Ultra				Norit CA1				Norit SX F Cat				AC Puriss			
adsorbent dose (mg/L)	AcCl^b^	DOC	UV_254_	SUVA	AcCl^b^	DOC	UV_254_	SUVA	AcCl^b^	DOC	UV_254_	SUVA	AcCl^b^	DOC	UV_254_	SUVA
10	92	40	5	0	80	20	5	0	86	32	5	0	91	38	41	1
25	94	42	42	6	94	29	9	0	95	38	27	0	95	41	45	3
50	95	49	63	32	98	42	5	0	92	41	36	0	92	58	68	21
75	93	54	79	57	>98	34	9	0	93	45	55	0	95	50	77	53
100	>98	62	84	61	>98	43	14	0	94	49	50	0	>98	66	86	58
200	>98	57	84	66	>98	51	27	0	94	54	77	28	>98	71	91	67
300	>98	62	89	74	>98	56	32	0	94	55	77	27	>98	62	95	88
500	>98	67	95	85	>98	62	36	0	94	59	86	49	>98	65	91	73
750	>98	70	95	84	>98	58	36	0	>98	61	82	32	>98	71	99	99
1000	>98	76	95	82	>98	66	50	0	>98	60	86	45	>98	72	99	99

	Norit SX Ultra				Norit CA1				Norit SX F Cat				AC Puriss			
adsorbent dose (mg/L)	MeCl^c^	DOC	UV_254_	SUVA	MeCl^c^	DOC	UV_254_	SUVA	MeCl^c^	DOC	UV_254_	SUVA	MeCl^c^	DOC	UV_254_	SUVA
10	>98	43	8	0	90	32	4	0	97	41	8	0	>98	40	58	0
25	>98	45	27	0	91	35	8	0	98	36	12	0	>98	43	73	16
50	>98	60	31	0	97	32	12	0	98	42	23	0	>98	47	85	44
75	>98	55	65	31	>98	36	15	0	>98	45	27	0	>98	51	88	66
100	>98	58	73	42	>98	38	27	0	>98	49	58	20	>98	56	88	72
200	>98	54	88	78	>98	49	38	0	>98	54	88	76	>98	60	92	69
300	>98	59	92	83	>98	55	46	0	>98	55	88	75	>98	52	92	77
500	>98	61	96	91	>98	58	54	0	>98	59	69	28	>98	67	98	81
750	>98	71	96	88	>98	59	56	0	>98	61	81	52	>98	63	98	93
1000	>98	71	96	88	>98	61	35	0	>98	60	85	63	>98	65	99	99

a All values represent the removal percentage of related parameters.

b AcCl: acetochlor.

c MeCl: metolachlor.

As adsorbent doses increased, the removal rates of NOM and UV_254_ also increased. The maximum NOM removal performances were 76, 66, 60, and 72% for acetochlor-containing samples and 71, 61, 60, and 65% for metolachlor-containing samples by Norit SX Ultra, Norit CA1, Norit SX F Cat, and AC Puriss, respectively. Although Norit CA1 had the lowest equilibrium capacity, it provided higher NOM removal performance than Norit SX F Cat at an adsorbent dose of 1000 mg/L. On the other hand, the highest and lowest UV_254_ removal performances were observed by AC Puriss and Norit CA1 with >99 and 50%, respectively. The fact that SUVA removal rates, especially at higher adsorbent doses, are higher than NOM removal efficiencies by the Norit SX Ultra and AC Puriss carbons indicates that aromatic and hydrophobic organic fractions are selectively removed by these carbons compared to hydrophilic and aliphatic organic substances. The selective removal of aromatic and hydrophobic organic fractions by the Norit SX Ultra and AC Puriss carbons might be attributed to the higher surface area of Norit SX Ultra and the higher total surface basic groups of AC Puriss. It is reported that the adsorption of amino and carboxylic groups on the adsorbent surface binds to the functional −OH group, and the number of total basic groups on the adsorbent surface affects the adsorption of organic compounds. As the amount of total basic groups on the adsorbent surface increases, the adsorption of organic compounds consisting of amino and carboxylic groups can be enhanced.^87^

In general, greater removal performances for acetochlor and metolachlor were obtained by all PACs, and both herbicides could be removed from the aqueous solution between 90 and >98% (Table 7). The highest acetochlor removal was observed by Norit SX Ultra, Norit CA1, and AC Puriss PACs, that is, >98% of acetochlor was adsorbed over 75 mg/L adsorbent dosages. However, an adsorbent dose higher than 500 mg/L should be applied in order to achieve over 98% acetochlor removal with Norit SX F Cat. On the other hand, metolachlor was almost completely removed by Norit SX Ultra and AC Puriss PACs, even at very low adsorbent dosages. Similarly, Norit CA1 and Norit SX F Cat provided higher metolachlor removal performances, and the results showed that it could be reduced to over 98% with higher than 50 mg/L doses.

The results were consistent with the study of Dai et al.,^88^ that is, acetochlor and metolachlor were removed successfully with the maximum efficiencies of 91.25 and 73.65% for 10 mg/L coal fly ash, respectively. Similarly, Wang et al.^7^ and Wang et al.^89^ reported 96.3 and >97.5% adsorption performances by the activated carbon and modified sorbent for acetochlor, respectively. In the metolachlor adsorption study with three different biochar pyrolyzed at different temperatures, removal performances varying between 15 and 91% were obtained depending on the pyrolysis degree of the biochar and the initial metolachlor concentration.^90^ Acetochlor and metolachlor are nonionic compounds, and they are characterized as less polar (3 < log *K*_ow_ < 4) by their log *K*_ow_ coefficients of 3.13 and 4.14, respectively (Table 1). The relatively higher adsorption efficiencies of metolachlor at a low adsorbent dose might be attributed to its higher log *K*_ow_ value than that of acetochlor, although their octanol–water partition coefficients are similar.

Besides, the nonionic nature of acetochlor and metolachlor caused the repulsion effect between the adsorbent and pollutant to be less significant, even though Norit CA1 is negatively charged at pH 7.85. Indeed, the possible sorption driving forces for nonionic pesticides like acetochlor and metolachlor are hydrogen and π–π bonds and van der Waals interaction rather than electrostatic interactions; therefore, the pH_pzc_ values of PACs did not have much effect on the adsorption behavior of pesticides.^91,92^ Even though they have a wide pH_pzc_ range, all adsorbents provided similar removal efficiencies, i.e., higher than 90% for acetochlor and metolachlor.

Adsorption studies have shown that for adsorbents having heterogeneous micropore size distribution, NOM competes with micropollutants dominantly by the direct site mechanism, and also primarily and the most favorable competition is the direct site for the NOM fraction, whose molecular size is closer to that of micropollutants.^93^ The lower SUVA removal observed at low adsorbent doses could possibly be associated with adsorption of low-molecular-weight, hydrophilic, and relatively aliphatic organics. Further, the relatively lower removal efficiencies for acetochlor and metolachlor at low adsorbent doses may be attributed to the readily occupied active adsorption sites of PACs by such organics. The results show that the low-molecular-weight NOM fraction is more competitive with acetochlor. On the other hand, a removal performance of over 98% was achieved even at the lowest dose in metolachlor adsorption by Norit SX Ultra and AC Puriss adsorbents. Similarly, Ling et al.^28^ reported that the inhibition of micropollutant adsorption was caused by the direct site competition of low- or medium-molecular-weight organics and/or pore blocking of high-molecular-weight organics. Kennedy and Summers^27^ stated that the significant reduction in adsorption capacities of methylisoborneol and warfarin was observed because of increasing direct site competition. On the other hand, Hu et al.^26^ observed that the low-molecular-weight hydrophobic NOM fraction was mostly responsible for competition in atrazine and caffeine adsorption. Wang et al.^7^ reported that humic acid, a component of NOM, competed with acetochlor for the adsorption sites of activated carbon functionalized with MnFe_2_O_4_, and the competition was more pronounced with the increase of humic acid concentration. Yu et al.^94^ stated that the removal of some fraction of NOM by coagulation pretreatment reduces the competition with the micropollutants against the adsorbent and increases the micropollutant removal efficiency.

## 4 Conclusions

In this study, we examined the removal of acetochlor, metolachlor, and NOM from surface waters with the low-SUVA characteristic by four different PACs (Norit SX Ultra, Norit CA1, Norit SX F Cat, and AC Puriss). Besides, the adsorption characteristics of PACs in terms of kinetic and isotherm models were evaluated.

The adsorption kinetic data of organic matter were well described by the PSO model for all tested activated carbons, with the kinetic rate constants changing between 0.19 and 0.39 g/mg-h. The chemical reaction or film diffusion was the rate-limiting step for adsorption. While the isotherm parameters well-obeyed the modified Freundlich isotherm, the highest NOM adsorption capacities were achieved by Norit SX Ultra with 120.6 and 127.2 mg/g and by AC Puriss with 99.5 and 100.6 mg/g for acetochlor- and metolachlor-containing samples, respectively.

Acetochlor and metolachlor were successfully removed with efficiencies in the range of 90–98% by all PACs, especially even at very low adsorbent dosages by Norit SX Ultra and AC Puriss. The possible mechanisms that occurred during adsorption of acetochlor and metolachlor by PACs were chemical and physical sorption, respectively. The adsorption affinity of PACs for acetochlor and metolachlor were in the order of Norit SX Ultra > AC Puriss > Norit CA1 > Norit SX F Cat and Norit SX Ultra > AC Puriss > Norit SX F Cat > Norit CA1, respectively. In general, the competitive effect of NOM on the adsorption of nonionic acetochlor and metolachlor, observed at low adsorbent dosages, indicates that low-molecular-size organic substances compete for the active sites of the adsorbent. Moreover, the inhibitory effect of NOM was more effective for acetochlor at low adsorbent doses for all PACs, while it was only observed for metolachlor in Norit CA. In practical applications, 100–200 mg/L Norit SX Ultra or AC Puriss can be applied for the efficient removal of acetochlor, metolachlor, and NOM at pH values of 7–8 for the treatment of waters like Altinapa surface water with NOM having low-molecular-weight organics and low-SUVA characters.

Future studies should also focus on the competitive effects of low-molecular-weight NOM fractions (e.g., neutral, hydrophilic, or hydrophobic acids and bases) and temperature effects on adsorption of nonionic pesticides by PACs and thorough investigation for nonionic pesticides’ physical and chemical interactions with PAC active sites.
